# Optimal Ancient DNA Yields from the Inner Ear Part of the Human Petrous Bone

**DOI:** 10.1371/journal.pone.0129102

**Published:** 2015-06-18

**Authors:** Ron Pinhasi, Daniel Fernandes, Kendra Sirak, Mario Novak, Sarah Connell, Songül Alpaslan-Roodenberg, Fokke Gerritsen, Vyacheslav Moiseyev, Andrey Gromov, Pál Raczky, Alexandra Anders, Michael Pietrusewsky, Gary Rollefson, Marija Jovanovic, Hiep Trinhhoang, Guy Bar-Oz, Marc Oxenham, Hirofumi Matsumura, Michael Hofreiter

**Affiliations:** 1 School of Archaeology and Earth Institute, Belfield, University College Dublin, Dublin 4, Ireland; 2 Department of Anthropology, Emory University, Atlanta, Georgia, United States of America; 3 independent researcher, Santpoort-Noord, The Netherlands; 4 Netherlands Institute in Turkey, Istiklal Caddesi, Nur-i Ziya Sokak 5, Beyoğlu, Istanbul, Turkey; 5 Peter the Great Museum of Anthropology and Ethnography (Kunstkamera), Russian Academy of Sciences, Universitetskaya Nab. 3, St. Petersburg, Russia; 6 Eötvös Loránd University University, Faculty of Humanities, Institute of Archaeological Sciences, Múzeum körút 4/b, Budapest, Hungary; 7 Department of Anthropology, University of Hawaii at Manoa, Honolulu, Hawaii, United States of America; 8 Whitman College, Walla Walla, Washington, United States of America; 9 Museum of Vojvodina, 21 000 Novi Sad, Ulica Dunavska 35, Serbia; 10 Institute of Archaeology, Hoan Kiem District, Hanoi, Vietnam; 11 Zinman Institute of Archaeology, University of Haifa, Mount Carmel, Israel; 12 School of Archaeology and Anthropology, Australian National University, Canberra, Australia; 13 School of Health Science, Sapporo Medical University, Sapporo, Japan; 14 Institute for Biochemistry and Biology, Faculty for Mathematics and Natural Sciences, University of Potsdam, Karl-Liebknechtstr. 24–25, 14476 Potsdam Golm, Germany; 15 Department of Biology, University of York, Wentworth Way, Heslington, York, United Kingdom; University of Oxford, UNITED KINGDOM

## Abstract

The invention and development of next or second generation sequencing methods has resulted in a dramatic transformation of ancient DNA research and allowed shotgun sequencing of entire genomes from fossil specimens. However, although there are exceptions, most fossil specimens contain only low (~ 1% or less) percentages of endogenous DNA. The only skeletal element for which a systematically higher endogenous DNA content compared to other skeletal elements has been shown is the petrous part of the temporal bone. In this study we investigate whether (a) different parts of the petrous bone of archaeological human specimens give different percentages of endogenous DNA yields, (b) there are significant differences in average DNA read lengths, damage patterns and total DNA concentration, and (c) it is possible to obtain endogenous ancient DNA from petrous bones from hot environments. We carried out intra-petrous comparisons for ten petrous bones from specimens from Holocene archaeological contexts across Eurasia dated between 10,000-1,800 calibrated years before present (cal. BP). We obtained shotgun DNA sequences from three distinct areas within the petrous: a spongy part of trabecular bone (part A), the dense part of cortical bone encircling the osseous inner ear, or otic capsule (part B), and the dense part within the otic capsule (part C). Our results confirm that dense bone parts of the petrous bone can provide high endogenous aDNA yields and indicate that endogenous DNA fractions for part C can exceed those obtained for part B by up to 65-fold and those from part A by up to 177-fold, while total endogenous DNA concentrations are up to 126-fold and 109-fold higher for these comparisons. Our results also show that while endogenous yields from part C were lower than 1% for samples from hot (both arid and humid) parts, the DNA damage patterns indicate that at least some of the reads originate from ancient DNA molecules, potentially enabling ancient DNA analyses of samples from hot regions that are otherwise not amenable to ancient DNA analyses.

## Introduction

The invention and development of next generation sequencing methods has resulted not only in a transformation, but also a rapid expansion of ancient DNA (aDNA) research. However, while substantial work has been conducted on optimization of ancient DNA extraction from bony specimens (e.g. [1–3]), which represent the vast majority of samples from which ancient DNA is extracted, less work has been done on the identification of the best skeletal elements with regard to aDNA preservation. Bearing in mind that DNA analyses are inherently destructive, it is necessary to identify, if possible, the skeletal elements most likely to yield utilizable and informative aDNA. However, no reliable methods for screening samples for DNA preservation are yet available [4]. While research has shown that the diagenetic status of the biomaterial from which aDNA will be extracted is correlated with the survival and preservation of DNA, the mechanisms of biomolecular deterioration as well as the location of DNA within the microscopic structure of the bone remain poorly understood [5]. Ultimately, when dealing with bone, the best indication that a sample has the potential to yield aDNA is to consider whether there is obvious evidence of microbial damage and to preferentially select dense areas of cortical bone. However, the relationship between macroscopic and molecular preservation still remains largely speculative [4]. In fact, most of the prevailing knowledge and assumptions about what skeletal parts are superior to others in terms of preservation of ancient DNA is based on forensic research [6]. The general notion in both forensic genetics and aDNA is that the density of a bone is positively correlated with DNA preservation and that sampling should be carried out whenever possible on dense, weight bearing bones, with a preference for the femur and tibia [7]. However recent studies by Gamba et al [8] and Rasmussen et al. [9] demonstrate the potential of the petrous portion of the temporal bone (‘petrous’ thereafter) as a region which provides high endogenous DNA yields. Additionally, the analysis of ancient hair samples, when available, has also yielded excellent endogenous DNA preservation (e.g. [10,11]). Endogenous DNA has also been successfully obtained from human teeth, including from the dentine [12]. Particularly high amounts of endogenous DNA have been extracted from the crushed cementum of the roots [13–15] and dental calculus is beginning to gain popularity as a substrate for the exploration of the oral microbiome and the impact on biocultural transitions in human history [16–18].

Leney [19] tested success rates of ancient mitochondrial (mt) DNA analyses of ~ 2,000 samples of human osseous and dental elements from casework of unidentified remains of U.S. service personnel from World War II, the Korean War, and the Vietnam War. Overall, differences in sample mass were found to be an important determinant of the probability of successful mtDNA recovery. However, some skeletal elements had high success rates independent of sample mass: cortical samples of dentine, femora, tibiae, mandibles, and first metatarsals. In contrast, cranial samples had low rates of DNA recovery. This demonstrates that the association between sample mass and DNA preservation is likely mediated by other factors. Edson et al. [20] examined sampling of different parts of the cranium in forensic cases at the Armed Forces DNA Identification Laboratory (AFDIL) and Defense POW/MIA Accounting Agency–Central Identification Laboratory for the analysis of mtDNA from the 558 cranial fragments tested from 1992 to 2009. They reported a statistically significant difference between the rates of success of the various cranial bones, in which the average success rates are 68% for the frontal bone, 65% for the occipital bone and 52% for the parietal bone. In contrast, the average success rates for the temporal bone were 90%. They also assert that in the case of the latter it is best to sample the inner, petrous portion, of the temporal bone which is less prone to weathering compared to other bones [21].

Climate of deposition and recovery was found to have only a minor effect on success rate in this study, with samples from temperate regions in general outperforming those from tropical regions. Of the 1,128 samples from tropical regions 66.9% yielded mtDNA while in the case of the 771 samples from temperate regions, the success rate was 75.9%, which is significantly larger. These results suggest that climate, while being a significant confounder, is not a major factor in rates of successful forensic mtDNA recovery [19]. However, it has been shown that environmental conditions are a key factor in long-term DNA survival [7], relevant for aDNA research. A number of environmental variables influence the rate of biomolecule degradation, including temperature, pH, the availability of oxygen, and exposure to water [22]. All other things being equal, the thermal history of a sample (considering both mean temperature and temperature fluctuations) is the key factor influencing DNA survival [7]. This is evident as many of the most successful aDNA studies utilized samples from permafrost regions (e.g. [7]). However, selection of samples from these areas is not always feasible and focusing on these regions introduces a major geographic bias with limited knowledge about aDNA from most of the world’s regions. Thus, especially when dealing with samples from warm climates, it is essential to extract DNA from the parts of the skeleton that preserve DNA best.

The petrous portion of the temporal bone (*pars petrosa*) is located at the base of the skull between the sphenoid and occipital bones, composing the endocranial part of the temporal bone that houses the delicate organs of hearing and equilibrium. The interconnected internal cavities of the petrous bone comprise the otic capsule of the inner ear [23]. This bony labyrinth consists of the cochlea, vestibule, and three semicircular canals. It is the hardest and densest bone part in the mammalian body [24]. The otic capsule, of particular interest to this study, develops from the cartilaginous differentiation of the mesenchyma encompassing the inner ear [25]. Ossification of the cartilage begins by the sixteenth week of gestation, the point at which the membranous labyrinth has reached its adult size, and involves fourteen centres that fuse to form a protective capsule surrounding the membranous labyrinth and perilymphatic space [26]. Ossification of the otic capsule is completed shortly before birth [26].

In a recent study, Gamba et al. [14] analysed complete and partial genomes sequenced from the petrous bones of 13 individuals from archaeological sites in Eastern Hungary, spanning from the Early Neolithic (~5,700 cal BC) to the Iron Age (800 cal BC). Additionally, they analysed the dentine for four individuals, ribs for two individuals, a metacarpal of one individual and a metatarsal of another individual, to compare endogenous yields from petrous bones to those from other skeletal/dental parts within the same individual. The endogenous DNA yields from the petrous samples exceeded those from the teeth by 4- to 16-fold and those from other bones up to 183-fold. In non-petrous bones and teeth, non-clonal endogenous DNA contents ranged from 0.3 to 20.7%, while the levels for petrous bones ranged from 37.4 to 85.4%. The authors suggested that the high endogenous yields obtained (>50% for 8 of the 13 specimens) is due to the high density of the petrous bone which is associated with reduced bacteria-mediated and other post-mortem DNA decay. However, density is not uniform throughout the petrous bone and it is necessary to investigate whether the high success rate is associated with bone density. In the case of one Neolithic individual (NE6) Gamba et al. [14] investigated two different areas of the same petrous bone: a more porous part and a denser part. They report that the denser part gave a higher endogenous yield (80.36%) when compared to the more porous part of the petrous (with an endogenous yield of 71.02%). These results pointed to the high potential of petrous bones for obtaining high endogenous yields both in humans and other species. However, it is not clear whether high yields can be obtained from any dense part of the petrous bone, hence suggesting that the high endogenous yields are associated with high bone density in the petrous, or rather with a particular anatomical part of the petrous, implying that the high yields are not just associated with bone density but with other anatomical and taphonomic aspects.

In this study, we further investigate the following questions, which are essential for the optimized analysis of ancient DNA from petrous bones:

1. Can optimal high yields be obtained from any dense part of the petrous bone? In particular can we detect a difference between the dense part of the inner ear (otic capsule) and other dense parts of the petrous?
2. Are there differences between dense parts in terms of average read lengths, damage pattern and total endogenous DNA concentration?
3. Is it possible to obtain high endogenous aDNA yields from petrous bones from hot environments, which are unfavorable for ancient DNA preservation?

## Materials

Ten petrous bones were selected from archaeological specimens, representing a wide range of geographical locations and climatic contexts (Table 1, for repository information see S1 File). The specimens were selected from Central Europe, Central Asia, Southeast Asia, the Levant, Anatolia, and North Africa. The specimens are from Holocene archaeological contexts dated to between 10,000–1,800 calibrated years before present (cal. BP). The samples from Nubia, Jordan and Turkmenistan are from hot and arid regions. The sample from Turkey is from the Eastern Mediterranean (northwestern Turkey); the samples from Hungary and Serbia are from the Carpathian Basin/Southeast Europe, while the two samples from Cambodia and Vietnam are from tropical/subtropical Southeast Asia. We also included a metatarsal bone for one Neolithic individual from Hungary (Polgár Ferenci hát, PF280-443) as a control to confirm the differences between petrous and non-petrous reported in the previous study [8].

**Table 1:** A summary table of the results obtained for the analysis of the A,B, C petrous bone parts for each of the ten specified samples.

Site	Country	Arch. Period	Age (BP)	Burial Number	A	B	C	MT
Parkhai II	Turkmenistan	Early Bronze Age	5000–4500	Grave 162, burial 61	0.10%	0.05%	0.04%	
Kulubnarti S	Egypt	Early Christian	2000	KulS5	0.12%	0.09%	0.04%	
Vat Komnou	Cambodia	Iron Age	2150–1750	40	0.22%	0.07%	0.27%	
Man Bac	Vietnam	Neolithic	3800–3500	M12	0.03%	0.04%	0.70%	
Ain Ghazal	Jordan	Pre-Pottery Neolithic	10000	AG93-CF 3883 burial 37	0.13%	0.11%	0.97%	
Barcın	Turkey	Pottery Neolithic	8400	L10E-106	3.37%	5.96%	45.24%	
Gomolava	Serbia	Middle Neolithic	6600	21–10	1.77%	49.45%	56.47%	
Polgar Ferenci hat	Hungary	Middle Neolithic	6300–6100	PF811/1144	0.20%	0.54%	35.36%	
Polgar Ferenci hat	Hungary	Middle Neolithic	6300–6100	PF145-253	0.49%	6.55%	49.58%	
Polgar Ferenci hat	Hungary	Middle Neolithic	6300–6100	PF280-443	42.00%	44.62%	69.63%	0.17%

A, B,C provide percentages of endogenous DNA contents. MT- metatarsal bone

## Methods

### Selection of sampling parts within the petrous bone

**Fig 1:**
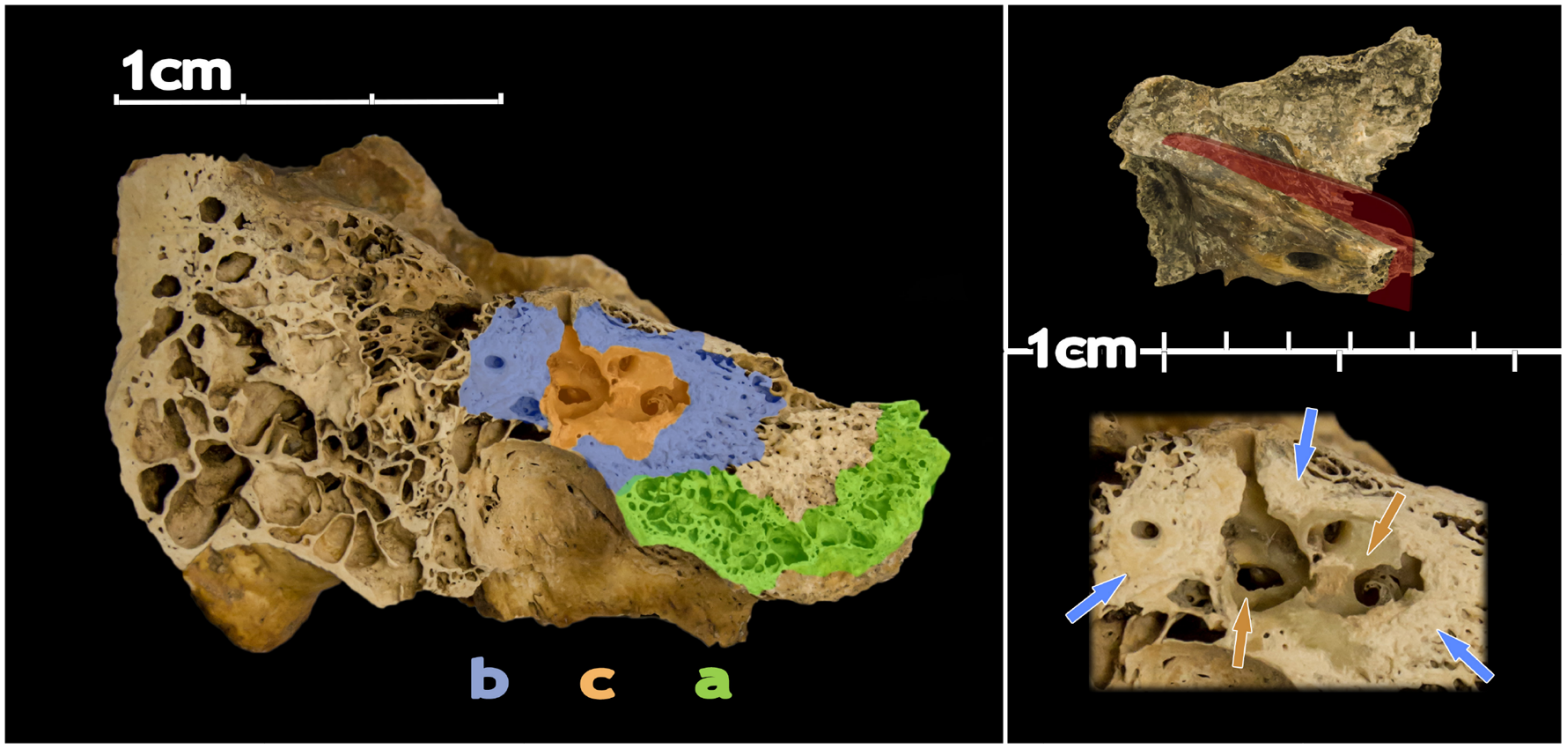
Medial view of a cut of a left petrous bone. The main image shows the location of the different areas targeted in this study (parts A, B and C) with different colours. The top box shows the direction of the cut. The lower box shows the area comprising parts B and C in detail and non-coloured. Blue and orange arrows point to areas of B and C, respectively.

In order to carry out intra-sample comparisons, we identified the three distinct areas for DNA extraction (Fig 1):

part A: bone at the apex of the petrous pyramid, which is largely trabecular (spongy).
part B: dense white bone, most commonly found surrounding the inner ear; depending on the preservation of the sample and natural variability (see S2 Fig) it can exist also in the area between the semi-circular canals, the outer ear, and the mastoid process.
part C: dense bone of the otic capsule (inner ear) which consists of the cochlea, vestibule, and three semi-circular canals, it surrounds the membranous osseous labyrinth and houses the organs of hearing and equilibrium in living organisms. In contrast to the whitish part B, it is of a yellowish-to-green range of hues.

While isolation and identification of part A is easily achieved due to the obvious porosity of the trabecular bone, separation of parts B and C requires precise work, since the inner ear (part C) is normally encapsuled in the dense white bone (part B). To isolate these parts, we combined the use of a Dremel disk saw and a sandblaster (Renfert Classic Basic). The latter allows for precise separation of the bone by controlling the output pressure, which in turn greatly helps in the identification of the inner ear (C) part. In attempting to identify part C, it is often easiest to first locate the superior semicircular canal before any sample processing occurs, which is easily identifiable on the unprocessed petrous bone by the arcuate eminence on the superior aspect of the bone.

In order to conduct intra-petrous comparisons on our archaeological samples, we first identified and isolated part A, and removed it from the rest of the petrous bone located in a UV cabinet. We then removed the dense white bone (part B) surrounding the otic capsule (part C) and then proceeded into clearing it of the remaining surrounding white bone (S1 and S2 Figs). All three parts were transferred to individual sample boats and put inside a UV chamber individually where they were decontaminated for 10 minutes on each side. Each part was then ground to very fine powder (~5 μm) using a mixer mill (Retsch MM400) and aliquots of 150 mg were recovered to proceed with DNA extraction. To minimize modern contamination, all these steps were done in a dedicated lab for preparation of ancient bone samples, with the researchers using full cover suits, double gloves, hair nets and face masks. All non-disposal equipment and work surfaces were cleaned and decontaminated with DNA-ExitusPlus and ethanol throughout the sample preparation process, and then subjected to UV radiation for at least 30 minutes.

### DNA extraction, library preparation and sequencing

DNA was extracted from each of the three petrous parts for 10 samples, and one metatarsal bone of the Hungarian specimen PF280-443, following protocol C from [27], a proteinase-K direct purification extraction protocol, as modified by MacHugh et al. [28] in a physically separated ancient DNA lab at University College Dublin. We included negative controls at a ratio of 1 control per 5 samples for all the involved steps: grinding, extraction, library preparation and indexing PCR. Approximately 150 mg of bone powder was suspended and digested in 1 mL of an extraction buffer solution containing TrisHCl (1M), sodium dodecyl sulfate (10%), EDTA (0.5M) and proteinase K (0.65U/mL) (Roche Diagnostics). Samples were incubated with rotation for 24h at 55°C, after which they were vortexed and re-incubated with rotation for another 24h at 37°C. A centrifugation step of 5 minutes at 17,000 g in a Heraeus Pico 17 microcentrifuge (Thermo Scientific) followed, separating the undissolved bone from the supernatant solution. The supernatant was collected and stored at -20°C, comprising the first extraction. The remaining bone powder was subjected to exactly the same extraction steps using a fresh extraction buffer, resulting in a second extraction. After another 48h, the second extraction supernatant was concentrated using Amicon Ultra-4 concentrators with a 30 kDa membrane (Millipore) to a final volume of approximately 100 μL. DNA from the second extraction was purified using the MinElute PCR purification kit (Qiagen) following the manufacturer’s instructions using EBT buffer prepared from the kit’s EB buffer (EB buffer plus TWEEN 20). These purified extracts were used for all subsequent library preparation.

Libraries for next-generation sequencing (NGS) were built with the DNA extracts using the protocol from [29], with a few modifications (see Supplementary Information). Indexing PCRs were performed using Accuprime Pfx Supermix (Life Technology), with primer IS4, and an indexing primer. 3 μL of the indexed library was added to a freshly prepared PCR mix, resulting in a total volume of 25 μL. PCR amplification was performed using the following temperature cycling profile: 5 min at 95°C, 12 cycles of 15 sec at 95°C, 30 sec at 60°C, and 30 sec at 68°C, followed by a final period of 5 minutes at 68°C. PCR reactions were then purified using the same MinElute (Qiagen) columns and methods as mentioned above. Assessment of the PCR reactions and concentrations of each sample were performed on the Agilent 2100 Bioanalyzer following the guidelines of the manufacturer (DNA concentrations can be found in Table 2). Based on the concentrations indicated by the Bioanalyzer, samples were pooled in equimolar ratios and sequenced on the Illumina MiSeq platform at the UCD Conway Institute of Biomolecular and Biomedical Research (University College Dublin, Ireland).

**Table 2:** DNA Concentration and estimated genome coverage obtained for the analysis of the A,B, C petrous bone parts for each of the ten specified samples.

Site	Powder Weigth (B, mg)			Endogenous yield			Concentration (ng/ul)			ng endog DNA/mg bone			# of genomes per total extract
	A	B	C	A	B	C	A	B	C	A	B	c	
Parkhai II	151	150	150	0.00	0.00	0.00	4.94	7.7	4.36	0.00	0.00	0.00	7
Kulubnarti S	146	152	149	0.00	0.00	0.00	7.78	11.2	12.54	0.00	0.00	0.00	12
Vat Komnou	151	150	147	0.00	0.00	0.00	0	16.49	13.21	0.00	0.00	0.00	158
Man Bac	153	151	145	0.00	0.00	0.01	12.57	9.81	8.42	0.00	0.00	0.01	507
Ain Ghazal	130	146	149	0.00	0.00	0.01	6.88	4.14	2.69	0.00	0.00	0.00	167
Polgar Ferenci hat	146	143	151	0.00	0.01	0.35	4.54	7.13	14.52	0.00	0.01	0.92	41516
Barcin	144	154	155	0.03	0.06	0.45	1.06	4.48	11.52	0.03	0.04	0.81	37582
Polgar Ferenci hat	150	151	152	0.00	0.07	0.50	7.32	4.38	8.89	0.02	0.05	0.74	33530
Gomolava	143	137	142	0.02	0.49	0.56	3.02	8.05	11.82	0.05	0.74	1.20	51251
Polgar Ferenci hat	151	149	151	0.42	0.45	0.70	2.02	4.59	12.25	0.74	0.37	1.50	68129

## Results

Bioinformatic analysis occurred via a customized python pipeline used to process the raw MiSeq data. Adapter sequences were trimmed using cutadapt v1.5 [30] with minimum overlap setting of 1 (- O 1) and minimum length at 17bp (-m 17). Reads were aligned to the human reference genome (hg19, GRCh37) using the Burrows-Wheeler Aligner [31] with disabled seed (-l 1000) and filtering for reads with a minimum QC score of 30, indicating 99.9% base call accuracy. Samtools v0.1.19-96b5f2294a [32] was used to remove duplicates. Damage patterns were assessed using the mapDamage tool [33] and normalized contamination estimates were calculated as in Gamba et al. [8]

**Fig 2:**
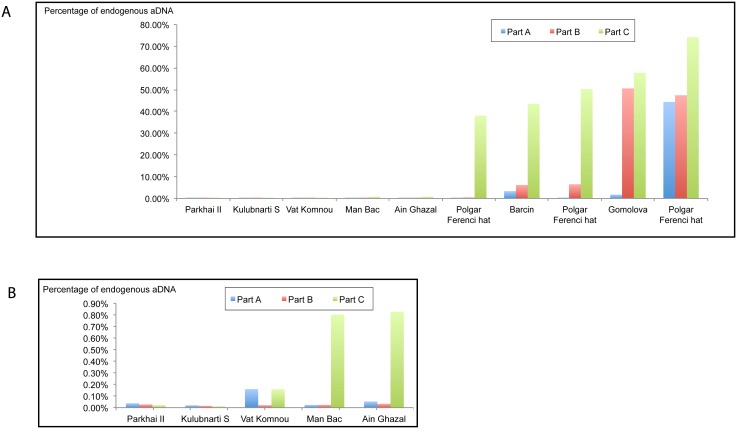
(A) Percentage of non-clonal human DNA recovered after shotgun sequencing for parts A, B and C for each of the ten specimens analysed, (B) Percentage of non-clonal human DNA recovered after shotgun sequencing for parts A, B and C for the five specimens analysed from hot, humid and arid parts.

We found substantial difference in both endogenous content and total endogenous DNA yields both between different petrous parts within samples and the same petrous part among samples (Table 1 and S2 File). The differences in yields for each of the ten specimens by bone sample type are shown in Fig 2a. Chi square analyses were carried out comparing percentages of endogenous content for the three parts for each of the ten specimens. In the case of each specimen the difference in endogenous content for parts A, B and C were significant at p <0.001. Five of the samples had maximum endogenous yields >35% (35.36%- 69.63%) for part C while the other five samples had average yields <1% for all parts. All the samples with low yields are those from tropical/subtropical (i.e. hot) regions. All five samples from temperate regions yielded high percentages of human DNA reads, at least for part C (Table 1). There is evidence of significant variation in inner ear yields between the three Neolithic samples from Polgár Ferenci hát. PF280-443 has relatively high yields for human DNA reads for all three petrous parts while PF145-253 has low yield for part A (0.49%), low-moderate yield for part B (6.55%) and high yield for part C (49.58%). PF811/1144 has low yield for part A (0.20%) low yield for part B (0.54%) and a high yield for part C (35.36%). A similar pattern was observed for specimen L10E-106 from the Neolithic site of Barcın, Anatolia, with low yield for part A (3.37%), low-moderate yield for part B (5.96%) and high yield for part C (45.24%). Finally, a different pattern was observed for specimen 21–10 from the Neolithic site of Gomolava, Serbia in which the yield for part A was low (1.77%), but high yields were obtained for part B (49.45%) and part C (56.47%). However, in all of the five cases in which some of the yields were high, the highest ones were always obtained for part C.

Following the analysis of percentage of endogenous DNA, we also examined variations between parts A, B and C in (a) damage patterns, (b) average read lengths, (c) and total endogenous aDNA yields.

For the temperate samples, there are, with one exception, no significant differences between the parts in damage patterns. Deamination patterns are consistent with the human reads representing endogenous DNA with elevated C-T substitutions at 3’ ends and elevated G-A substitutions at 5’ ends [34] for all three parts from all five samples, except for part A from the Hungarian sample PF811/1144.

**Fig 3:**
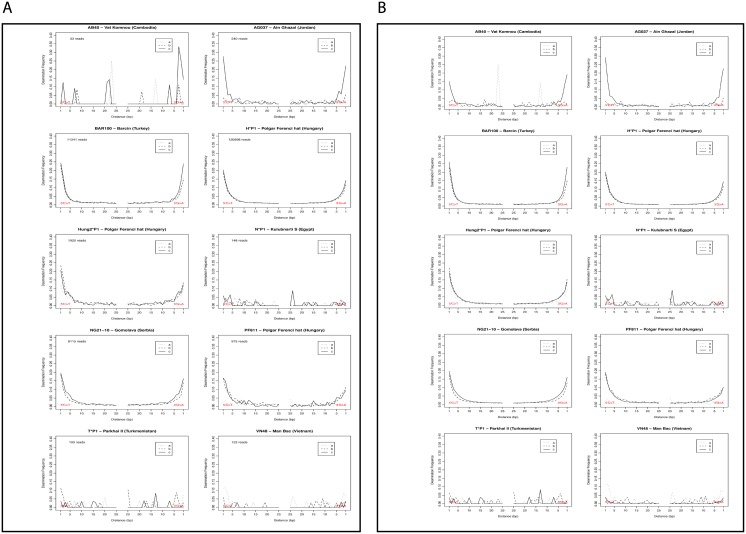
(A) Deamination patterns for each of the cases for bone parts A, B and C, before subsampling, (B) Deamination patterns for each of the cases for bone parts A, B and C, after subsampling.

Of the five samples from hot regions with low numbers of reads mapping to the human genome, the one from Man Bac, Vietnam has a much higher percentage of endogenous yields in part C (0.7% vs. 0.03% for part A and 0.04% for part B). A similar pattern is evident in the case of Ain Ghazal with 0.97% endogenous yield for part C vs. 0.13% for part A and 0.11% for part B (Fig 2b). However, human DNA read numbers are too low to compare deamination patterns for the reads obtained from different parts of the samples from hot environments, except for part C from samples Man Bac, Ain Ghazal and Vat Komnou. For the latter two of these samples, the deamination patterns for reads from part C show > 30% deaminated cytosines at both ends of the DNA fragments, strongly suggestive that the majority of these sequence is ancient, and therefore authentic [19]. In contrast, the deamination pattern for the results obtained from part C of the sample Man Bac, 3800–3500 cal. BP, does not match what is generally expected for endogenous ancient DNA sequences, although it shows a slight elevation of deamination at either end of the reads (Fig 3a). Moreover, the human reads for part C from this sample are also substantially longer than the human reads from the other two parts, but also longer than the non-aligned (most likely microbial) reads obtained from this sample. Thus, the human sequencing reads in this case could well be the result of contamination, despite extremely low estimated modern contamination for part C (0.09%) (S2 File). The only other significant differences in read lengths between parts from individual samples were found for sample Vat Komnou (AB40 Cambodia). Here, reads from part A are substantially longer than reads from parts B and C. However, there are so few human reads from part A (33) that it is not possible to calculate deamination patterns for this part, as also shown by subsampling the reads from the other two parts down to 33 reads (Fig 3b). In contrast, using all mapping reads especially part C shows high deamination, with between 20 and 30% on both ends of the sequenced fragments.

We also found substantial differences between bone parts with regard to total endogenous DNA yields. Thus, parts A and B yield maximally 0.73 ng DNA per mg bone, while part C yields maximally 1.41 ng DNA / mg bone (Table 2). Moreover, for the five temperate bones, part C yields between 1.6 and >126 times more total endogenous DNA per mg of bone than part B and between 2 and > 109 times more than part A. Finally, if we assume all human reads are of endogenous origin—an assumption that is more likely for the temperate than for the tropical bones, and therefore resulting in a conservative estimate—the total amount of endogenous DNA per mg of bone differs by four orders of magnitude among bones (Table 2), emphasizing the importance of environmental condition for long-term DNA preservation.

Endogenous yield for the metatarsal bone from specimen PF280-443 is 0.17% (Table 1) and hence the increase in endogenous content when comparing this result to those obtained for the petrous bone of the same individual (parts A, B, and C) is as much as 410-fold. However, even including the samples from [8], the number of comparisons between petrous and non-petrous bones is relatively small. Therefore, the difference in endogenous content may well be even larger for some samples.

## Discussion

It has recently been demonstrated [8] that petrous bone samples yield exceptionally high percentages of endogenous ancient DNA. Here we have shown that both the total amount of endogenous DNA that can be recovered as well as the percentage of all reads that represents endogenous DNA vary substantially for different parts of the petrous bone. Our results have several implications for aDNA studies. The results support the hypothesis that dense bone parts are especially suitable for ancient DNA research, with the densest part of the petrous bone, that which composes the otic capsule, providing the best results. For our samples the yields obtained for this part (part C) exceed those obtained for part B (i.e. dense bone part of the petrous outside the otic capsule) by up to 65-fold and those from part A by up to 177-fold. It is therefore apparent that while high endogenous yields can be obtained from part B, and hence from any dense part in the petrous, optimal yields should be obtained from bone sample taken directly from the otic capsule.

The inner-ear bone (part C) is affected by inhibition of bone resorption towards the perilymphatic space. Animal studies with osteofluorochrome labelling seem to confirm that the remodelling rate in this region is low and suggest that bone remodelling is progressively inhibited toward inner-ear spaces where the labelling is increasingly sparse [24]. Sorensen et al. [35] assessed the distribution of bone remodelling inside the otic capsule by analysing the bone histology of undecalcified temporal bones from adult rabbits labelled in vivo with bone-seeking fluorochromes. Results indicate that there is an increase in the number of bone remodelling units in relation to their centrifugal distance from the perilymphatic space in the otic capsule. This suggests that remodelling control affects the entire bony otic capsule and that this effect is reduced in the case of the outer capsular parts. However, to our knowledge, no absolute values for bone turnover inside the otic capsule or, more generally, for the temporal bone exist and without such data it is not possible at this stage to assess whether there is a relationship between turnover rates and endogenous aDNA yields in petrous bones.

The difference in endogenous yields for the single non-petrous bone in our sampling, the metatarsal bone from specimen PF280-443 (0.17%, Table 1), compared to petrous samples from the same individual is between 267- and 410-fold and hence even larger than the maximum difference reported in Gamba et al [8] in which the largest difference between a petrous and non-petrous bone was 186-fold when contrasting the endogenous yield from a rib and a petrous bone of individual IR1. Although it represents only a single data point, the result shows that extremely poor endogenous DNA yields from non-petrous bones of a sample do not exclude high endogenous DNA yields from the petrous bone of the same specimen. It seems that this micro-part is not affected by the taphonomic processes in the same manner as other parts of the skeleton, as it is encased in dense bone which is located inside the skull. This result also suggests that in cases when no petrous bone is available—either because this part is missing from the skeleton or when working on species other than mammals, such as birds or reptiles—researchers should try to use other dense bones for ancient DNA studies. Our analyses further suggest that there is a difference in aDNA concentration (ng/μl) between part C and parts A and B. Moreover, for bones from temperate environments, relatively small amounts of bone (in the low mg range) contain sufficient amounts of endogenous DNA to allow for multifold coverage sequencing of the nuclear genome. Thus, it is less the total amount of bone used but rather the choice of the right part of bone that is key for successful palaegenome projects, at least with samples from temperate environments.

Finally, our results show that endogenous yields from the five samples which originated from hot (either arid or humid) regions were always lower than 1% including extractions from part C of the petrous bone. However, deamination patterns suggest for two (Ain Ghazal and Vat Komnou) of the three samples for which we obtained sufficient numbers of reads that the obtained sequences are likely endogenous to the bones (S3 Fig). In contrast, the deamination pattern for the third sample, Man Bac, suggests that the human reads obtained are more likely to represent contamination than endogenous ancient DNA. These results suggest that it may be possible to obtain endogenous DNA from part C also for samples with relatively low amounts of endogenous DNA from hot environments, although extreme caution will be necessary in the interpretation of the results obtained from such samples. Thus, samples from such environments remain problematic for ancient DNA research even when choosing the part of the skeleton with best ancient DNA preservation. However, the fact that Man Bac and Ain Ghazal samples, which are both from regions with environmental conditions which are unfavorable to aDNA preservation, yielded endogenous human DNA (although in low percentages), is encouraging as it suggest that in the future, analyses of petrous bones from regions such as sub-Saharan Africa, the Middle East and Southeast Asia, may provide sufficient nuclear DNA data to carry out genomic-scale analyses, although currently still at high costs. Further experiments involving additional samples and deeper sequencing will be necessary to investigate the potential of petrous bone sampling from hot regions, but given the great interest in human ancient DNA from these regions, such studies are well-warranted.

## Supporting Information

S1 File Repository Information and permits for the specimens analysed in this study (XLSX)

S2 File Sequencing data and contamination estimates based on tests in Gamba et al. [8]. (XLS)

S1 Fig The inner ear with white dense bone (I) and without (II). Blue arrows point to white dense bone (B) and orange arrows to the differently coloured bone of the inner ear (C). In II, some areas still contain white dense bone due to difficulties in removing it while maintaining the semi-circular canals.
(TIF)

S2 Fig Density variability in Neolithic petrous bones. Left and right bones are from a Neolithic site in Hungary (6300–6100 BP) and the middle bone from a Neolithic Croatian site (5000–4000 BC).
(TIF)
