# Supplementary figures and images for: Optimal Ancient DNA Yields from the Inner Ear Part of the Human Petrous Bone
Source: PLoS One. 2015 Jun 18;10(6):e0129102. doi: 10.1371/journal.pone.0129102 (PMC4472748; doi:10.1371/journal.pone.0129102)

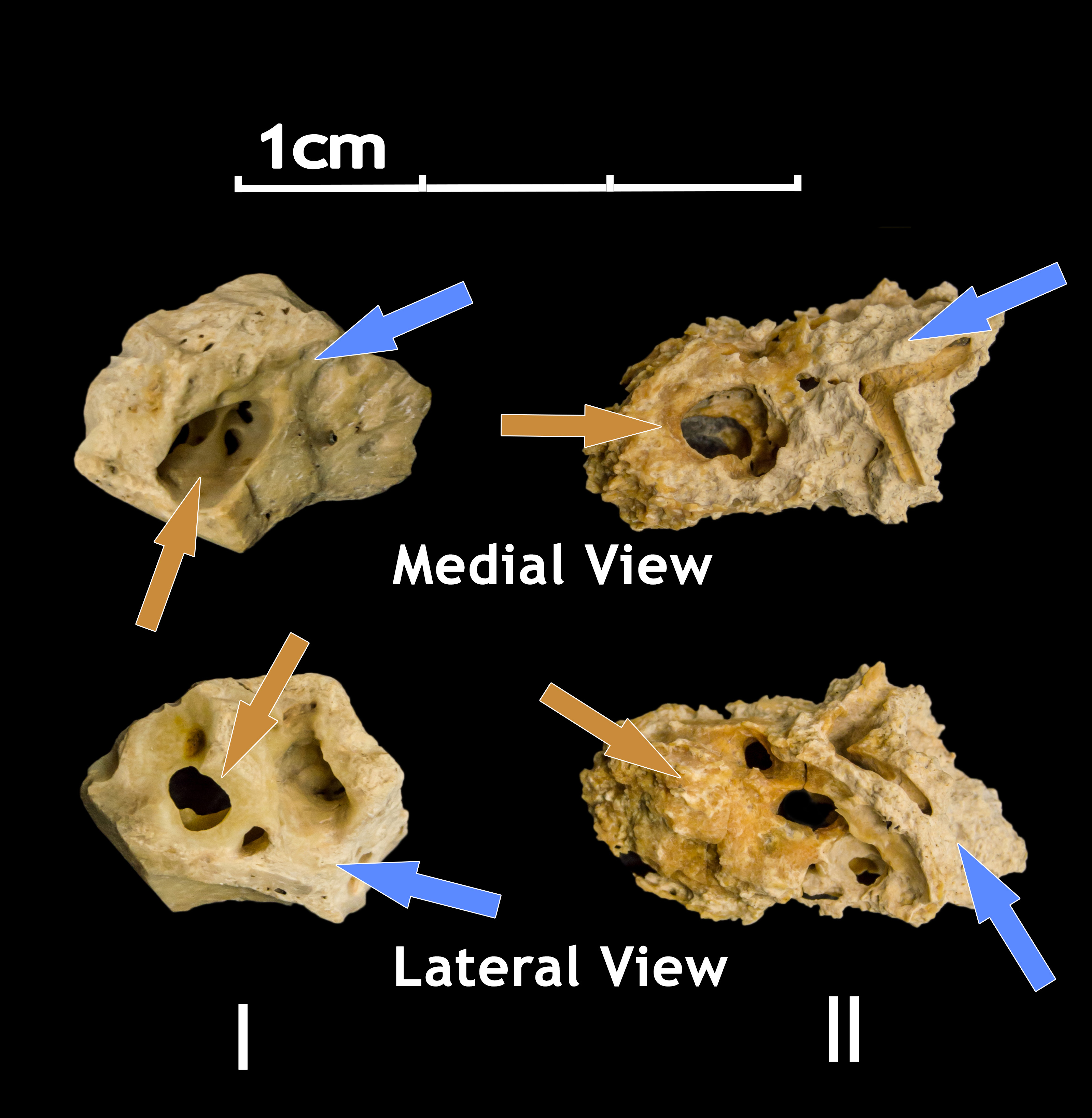

Supplement: S1 Fig — Blue arrows point to white dense bone (B) and orange arrows to the differently coloured bone of the inner ear (C). In II, some areas still contain white dense bone due to difficulties in removing it while maintaining the semi-circular canals. (TIF) [file pone.0129102.s003.tif]

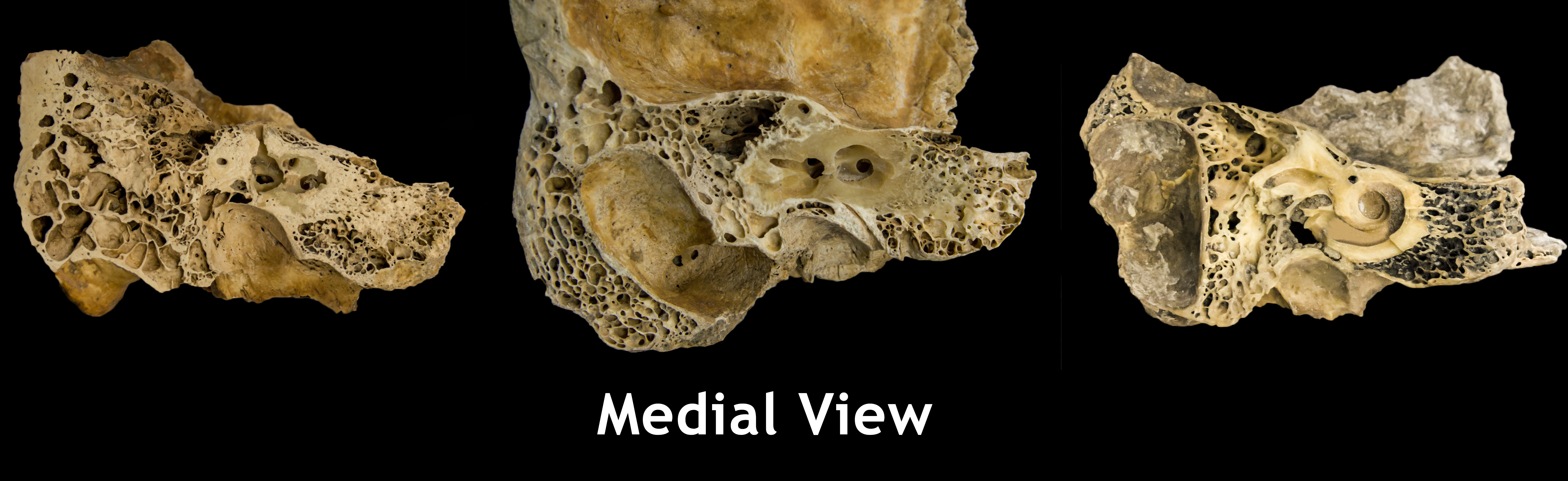

Supplement: S2 Fig — Left and right bones are from a Neolithic site in Hungary (6300–6100 BP) and the middle bone from a Neolithic Croatian site (5000–4000 BC). (TIF) [file pone.0129102.s004.tif]
